# Fresnel diffractograms from pure-phase wave fields under perfect spatio-temporal coherence: Non-linear/non-local aspects and far-field behavior

**DOI:** 10.1038/s41598-017-17493-w

**Published:** 2017-12-18

**Authors:** F. Trost, S. Hahn, Y. Müller, S. Gasilov, R. Hofmann, T. Baumbach

**Affiliations:** 1 Laboratorium für Applikationen der Synchrotronstrahlung, Karlsruher Institut für Technologie, Kaiserstr. 12, D-76131 Karlsruhe, Germany; 2 Institute for Photon Science and Synchrotron Radiation, Karlsruher Institut für Technologie, Hermann-von-Helmholtz-Platz 1,, D-76344 Eggenstein-Leopoldshafen, Germany

## Abstract

Recently, the diffractogram, that is, the Fourier transform of the intensity contrast induced by Fresnel free-space propagation of a given (exit) wave field, was investigated non-perturbatively in the phase-scaling factor *S* (controlling the strength of phase variation) for the special case of a Gaussian phase of width $\sqrt{{\bf{w}}}$. Surprisingly, an additional low-frequency zero σ_*_ = σ_*_(*S*, *F*) >0 emerges critically at small Fresnel number *F* (σ proportional to square of 2D spatial frequency). Here, we study the S-scaling behavior of the entire diffractogram. We identify a valley of maximum S-scaling linearity in the *F* − σ plane corresponding to a nearly universal physical frequency *ξ*ml = (0:143 ± 0.001)*w* ^−1/2^. Large values of *F* (near field) are shown to imply *S*-scaling linearity for low *σ* but nowhere else (overdamped non-oscillatory). In contrast, small *F* values (far field) entail distinct, sizable s-bands of good *S*-scaling linearity (damped oscillatory). These bands also occur in simulated diffractograms induced by a complex phase map (Lena). The transition from damped oscillatory to overdamped non-oscillatory diffractograms is shown to be a critical phenomenon for the Gaussian case. We also give evidence for the occurrence of this transition in an X-ray imaging experiment. Finally, we show that the extreme far-field limit generates a σ-universal diffractogram under certain requirements on the phase map: information on phase shape then is solely encoded in *S*-scaling behavior.

## Introduction

The emergence of intensity contrast through free-space propagation of a phase modulated paraxial wave field^1,2^ is exploited in many imaging modalities including neutron scattering, transmission electron microscopy, and X-ray phase-contrast imaging. When the exit wave field is dominated by phase modulations, e.g. for hard X-rays and samples composed of low-*Z* elements^3–9^, propagation based phase contrast (PBPC) essentially is nearly pure (very little absorption of the impinging beam by the sample) and therefore dose efficient. In the context of X-ray PBPC, this implies that e.g. 4D *in vivo* imaging of early-stage vertebrate-model embryos can be performed over several hours at time lapses of about ten minutes^10^. In^11–13^ quasi-particle (QP) phase retrieval from single-distance intensity contrast was proposed for projected **m**ulti-scale **o**bjects of a **b**road **s**pectrum (MOBS). This method appeals to an overall linear *S*-scaling of the intensity contrast *g*_*z*_ ≡ (*I*_*z*_−*I*_0_)/*I*_0_ where *I*_0_, *I*_*z*_ denote the intensities at object exit and at a downstream-propagation distance *z*. For 0 ≤ *S* ≤ *S*_*c*_ exceptional thin spectral regions in the diffractogram, which violate this linear behavior, can justifiably be neglected. Here *S*_*c*_ denotes a critical value of *S* where non-linear *S*-scaling starts to become a global spectral feature. In practice *S*_*c*_ rarely is reached. The term *S*-scaling refers to the response of the diffractogram under a scaling of the phase map, *ϕ* → *Sϕ* (*S* > 0), and the virtue of diffractograms is that they allow for simple phase retrieval in models with linearized phase-to-intensity contrast transfer due to certain linear differential position-space operators acting purely algebraically.

It was shown in^13^ that quasi-particle phase retrieval, which still is algebraic, boosts spatial resolution and dose efficiency if employed at large propagation distances *z*. The latter are feasible experimentally with, e.g., highly coherent hard X-rays produced by 3rd-generation synchrotron sources and free-electron lasers^14^.

The present work continues to pursue an investigation initiated in^15^. There, the diffractograms, induced by phase maps representing projected **s**ingle-scale **o**bjects of a **b**road **s**pectrum (SOBS), were analysed by semi-analytical continuum methods (no appeal to a pixelization). Even though these phase maps may seem to be an over-simplification of realistic object projections their investigation allows for certain fundamental and generic insights into non-linear effects in Fresnel theory. Concerning direct applications, the far-field diffractograms induced by SOBS phase maps could be of interest to self-interferometrically infer the sizes of astronomical objects. In^15^ the low-frequency regime in such diffractograms was investigated for a perfectly spatio-temporally coherent beam of wavelength *λ*. Based on the well-controlled non-locality expansion^15^ an unexpected occurrence of a zero σ_*_ in addition to the zeros predicted by the linear order in *S* was found. Here

1 $$\sigma \equiv \pi \lambda z{\xi }^{2},$$

and *ξ* denotes the vector of 2D of spatial frequency. This non-linearly generated zero σ_*_ rises critically from 0 to finite values with increasing *S* or decreasing *F* ≡ *w*/(*λz*). Here, $\sqrt{w}$ denotes the width of the Gaussian exit phase map

2 $${\varphi }({\bf{x}})=S\exp (-\frac{{{\bf{x}}}^{2}}{2w})\equiv S\exp (-\frac{{r}^{2}}{2w}),$$

where **x** ≡ (*x*, *y*)^*T*^ indicates the 2D position-space vector in the plane perpendicular to the optical axis. One goal of the present work is to identify the spectral location of maximal *S*-scaling linearity throughout the entire spectrum. Moreover, we address, in dependence of *F*, a critical phenomenon describing a sharp transition between two alternative “states” of the diffractogram: damped oscillatory vs. overdamped non-oscillatory. In addition, we investigate σ-universal behavior of diffractograms in the extreme far-field limit in the sense that phase-shape information transmutes into *S*-scaling behavior. We mainly base this analysis on the Gaussian phase map (2) since it grants a semi-analytic, continuum treatment. However, certain spectral features turn out to be generalizable to the case of more complex phase maps. Exemplarily, we demonstrate this by also analysing pixelated simulated and experimental data.

This paper is organized as follows. Sec. 1 reviews the low-σ results on Gaussian-SOBS induced diffractograms obtained in^15^ to lay the basis for the present work and to provide useful benchmarks. For the phase map of Eq. (2) and at a given value of σ we determine in Sec. 2 the value of *F* such that the diffractogram exhibits maximal *S*-scaling linearity (ml) within a prescribed scaling window 0 ≤ *S* ≤ *S*_*u*_. It turns out that the associated frequency modulus |*ξ*|_ml_ is nearly independent of *F*: It only depends on *w*. In Sec. 2.2 we also analyse σ-bands of approximately linear *S*-scaling and notice that their widths depend on *F*: In the near field (large *F*) only a single such band, including σ = 0, exists whereas the far-field situation (small *F*) exhibits several bands, excluding the point σ = 0. This motivated the characterization of a global spectral and *critical* transition from a damped oscillatory to an overdamped non-oscillatory diffractogram under an increase of *F*. In Sec. 2.4 we investigate bands of *S*-scaling linearity in simulated (pixelated) diffractograms, induced by a more complex phase map. Also, we demonstrate the transition from a damped oscillatory to an overdamped non-oscillatory diffractogram in experimentally generated diffractograms. Sec. 3 addresses the extreme far-field limit. First, we investigate the diffractograms induced by three different SOBS phase maps which become re-scaled Dirac delta functions for *w* → 0. While the σ dependence of these diffractograms universally resides in cosine and sine functions, phase-shape specific information exclusively is encoded in their *S*-scaling behavior, see Sec. 3.1. These results have motivated an according investigation of all those MOBS phase maps which become re-scaled delta functions in the limit where their spatial scales {*w*_*i*_} homogeneously approach zero. Again, phase maps in this class induce σ-universal diffractograms. Sec. 3.2 presents a brief summary of our results.

## State of the art: SOBS diffractograms at low frequencies

In^15^ the non-locality expansion of the diffractogram as a useful approximation scheme for localized phase maps was developed. Namely, it considers the phase-overlap product

3 $${{\varphi }}_{+}{{\varphi }}_{-}\equiv {\varphi }({{x}}_{\perp ,+}){\varphi }({{x}}_{\perp ,-}),$$

where **x**_±_ ≡ **x **± *λzξ*/2, to be considerably smaller than *S* ^2^. This suggests the expansion of the mutual intensity in the exit plane into powers (*ϕ*_+_ *ϕ*_−_)^*l*^ (*l* = 0, 1, 2, …), see right-hand side of Eq. (4). Notice that no assumption on the smallness of *S* is being made. Truncating this expansion at higher and higher values of *l* allows for improved control of lower and lower σ-values in the diffractogram which then increasingly represent non-local effects. That is, the zeroth order in this expansion (zeroth “onion shell”) is purely local and dominates the far-field diffractogram for all σ > 0. Technically, the non-locality expansion is based on Guigay’s important representation of the induced intensity’s Fourier transform (${\mathcal F}$) in terms of the “Fourier transform” of the exit wave field’s mutual intensity^16^:

4 $$ {\mathcal F} {I}_{z}=\int {{\rm{d}}}^{2}x{\psi }_{0}({{\bf{x}}}_{-}){\psi }_{0}^{\ast }({{\bf{x}}}_{+})\exp (-2\pi {\rm{i}}\xi \cdot {\bf{x}}),$$

where ${\psi }_{0}({\bf{x}})=\sqrt{{I}_{0}}\exp [{\rm{i}}{\varphi }({\bf{x}})]$ denotes the (pure-phase) exit wave field, and perfect spatio-temporal coherence is assumed. For the Gaussian phase of Eq. (2) the normalized diffractogram reads

5 $$\frac{ {\mathcal F} {g}_{z}}{4\pi w}\equiv \hat{g}(\sigma ,F,S)\equiv \sum _{l=0}^{\infty }{\hat{g}}_{l}(\sigma ,F,S)=\frac{1}{2}\sum _{n=1}^{\infty }\sum _{l=0}^{n}\frac{{({\rm{i}}S)}^{n}}{n}\frac{{(-\mathrm{1)}}^{l}}{l!(n-l)!}\exp [{D}_{n,l}(\sigma ,F)],$$

where

6 $${D}_{n,l}\equiv -(\frac{2\pi F}{n}+\frac{n}{2\pi F}[\frac{l}{n}-\frac{{l}^{2}}{{n}^{2}}])\sigma -{\rm{i}}\frac{n-2l}{n}\sigma \mathrm{.}$$

For *F* → 0 the sum over *l* in Eq. (5) reduces to the contribution of *l* = 0 and *l* = *n*, ${\hat{g}}_{l=0}(\sigma ,F,S)$ (zeroth onion shell), if σ > 0. Then one has^15^

7 $$\begin{array}{rcl}\mathop{\mathrm{lim}}\limits_{F\to 0}\hat{g}(\sigma ,F,S) & = & \mathop{\mathrm{lim}}\limits_{F\to 0}{\hat{g}}_{0}(\sigma ,F,S)={\rm{SI}}(S)\,\sin (\sigma )+{\rm{CI}}(S)\,\cos (\sigma )\\  & = & \sqrt{{{\rm{CI}}}^{2}(S)+{{\rm{SI}}}^{2}(S)}\,\sin (\sigma -{\sigma }_{s})\,,\end{array}$$

where

8 $${\rm{SI}}(S)\equiv {\int }_{0}^{S}{\rm{d}}x\,\frac{\sin (x)}{x}\,,{\rm{CI}}(S)\equiv {\int }_{0}^{S}{\rm{d}}x\,\frac{\cos (x)-1}{x},$$

and

9 $${\sigma }_{s}(S)\equiv |\arctan (\frac{{\rm{CI}}(S)}{{\rm{SI}}(S)})|\,\mathrm{.}$$

It is clear that with increasing σ the approximation $\hat{g}(\sigma ,F,S)\sim {\hat{g}}_{0}(\sigma ,F,S)$ is increasingly accurate at finite *F*, compare with Eq. (5).

**Figure 1:**
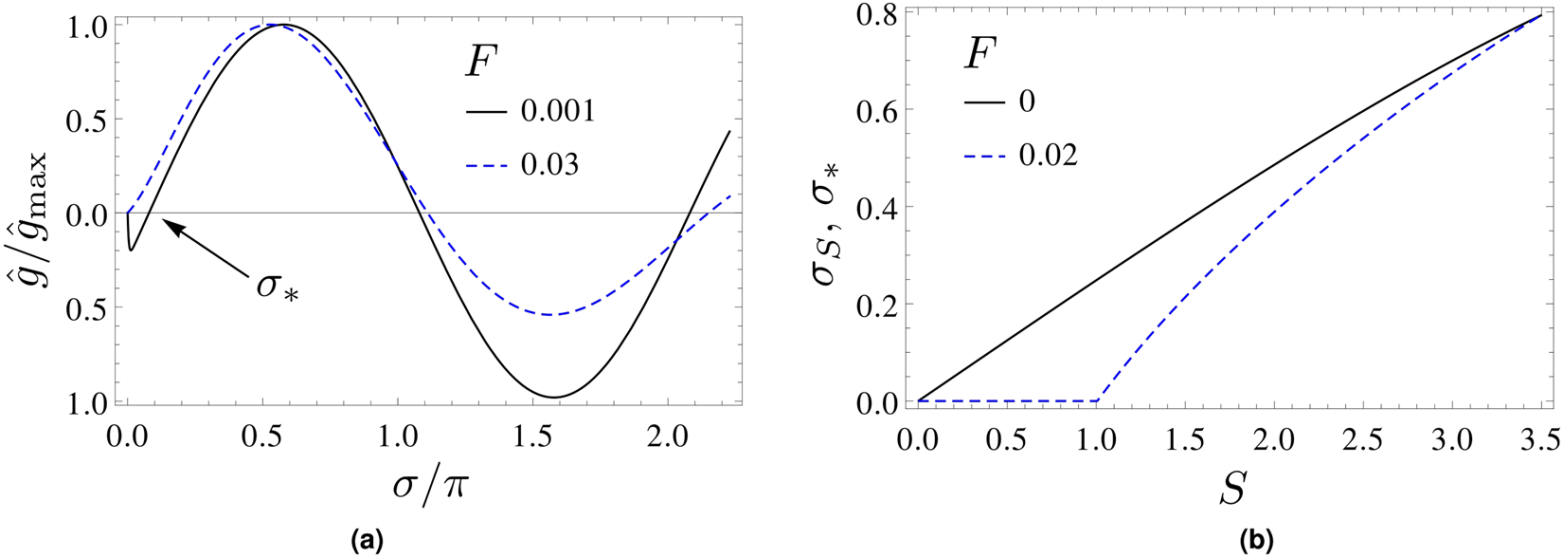
Low-frequency region of normalized diffractogram $\hat{g}(\sigma ,F,S)$ as induced by the Gaussian phase of Eq. (2). (**a**) $\hat{g}(\sigma ,F,S)$ for *F* = 0.001 (solid, overcritical) and *F* = 0.03 (dashed, subcritical) at *S* = 1. Note the additional low-frequency zero σ_*_ in the overcritical case. (**b**) *S*-dependence of σ_*_(*S*, *F* = 0) = *σ*_*s*_(*S*) (solid) and σ_*_(*S*, *F* = 0.02) (dashed). Note the non-differentiability of the latter curve at $S=16\pi F\sim 1.0$.

Flux conservation as implied by free-space propagation demands that $\hat{g}(\sigma =\mathrm{0,}\,F,S)=0$ for any value of *F* and *S*. Compared to the contrast-transfer-function (CTF) model, obtained by linearization of ${\mathcal F} {g}_{z}$ w.r.t. *S* ^9^, an additional zero σ_*_(*S*) = σ_*s*_(*S*) exists for *F* → 0 which is located in between σ = 0 and σ = π. At finite *F* the additional zero σ_*_(*S*) may or may not be vanishing, depending on whether the point (*S*, *F*) is to the left (subcritical) or right (overcritical) of the line

10 $$F=\frac{S}{16\pi }$$

in the *S*-*F* plane^15^, see Fig. 1a,b: traversing the line of Eq. (10) from left to right (*S*-transition) or top to bottom (*F*-transition), σ_*_ opens up critically like an order parameter in a second-order phase transition. The respective critical exponents neither depend on *F* nor *S* ^15^. We have noted that in Appendix D of^15^ four typos have occurred. One should replace i^*n*^ → (−i)^*n*^ in Eq. (D2), ${(-{\rm{1}})}^{n}\to {(-{\rm{1}})}^{l}$ in Eq. (D3) and ${(-{\rm{1}})}^{k+l}\to {(-{\rm{1}})}^{k+l+1}$ in Eq. (D8).

## SOBS: *S*-scaling linearity and global spectral transition

In this section we investigate additional spectral aspects of *S*-scaling within Gaussian SOBS diffractograms. Namely, in Sec. 2.1 we design a quantity *D*(σ, *F*) to measure deviations from linear *S*-scaling at prescribed values of σ and *F* within a window $0\le S\le {S}_{{\rm{\max }}}={\mathscr{O}}(1)$. It turns out that *D*(σ, *F*) possesses a line of almost degenerate minima (floor of a valley) in the σ-*F* plane. This function can be inverted and fits well to a power-law model σ(*F*) = *AF* ^*B*^. Since *B* ≈ −1 we infer from the definition σ ≡ *πwξ* ^2^ *F* ^−1^, compare with Eq. (1), that the physical frequency modulus, *ξ*_ml_, where maximal *S*-scaling linearity occurs, is *independent* of *F*. This result could be of relevance in applications because it allows to extract the width $\sqrt{w}$ by identifying one and the same point *ξ*_ml_ in non-locally dominated diffractograms corresponding to a range of propagation distances. In Sec. 2.2 the concept of spectral bands of approximate *S*-scaling linearity is introduced and applied to diffractograms in dependence on *F*. Again, an emergent linearity at low frequencies and large *F* can be pinned down which arises due to non-local effects. Moreover, in Sec. 2.3 we investigate a global and sharp spectral transition in the diffractogram from damped oscillatory to overdamped non-oscillatory. The order parameter *k*_max_ for this transition–the (pseudo) frequency of oscillation–drops critically at the value *F*(*S*) like the one in a second-order phase transition. Finally, in Sec. 2.4 we verify the usefulness of spectral bands of approximate *S*-scaling linearity in analysing simulated diffractograms as induced by more complex phase maps. Also, we provide experimental evidence for the transition from damped oscillatory to overdamped non-oscillatory as identified in Sec. 2.3 for the case of Gaussian SOBS diffractograms.

### Scaling properties of low-frequency diffractogram in dependence of *F*

To design a measure for the linearity in *S*-scaling of $\hat{g}(\sigma ,F,S)$ at given values of σ and *F* (Gaussian phase map of Eq. (2)), we define the function *D*(*S*_*u*_, σ, *F*) as

11 $$D({S}_{u},\sigma ,F)\equiv \frac{\sqrt{{\int }_{0}^{{S}_{u}}{|\frac{\hat{g}(\sigma ,F,{S}_{u})}{{S}_{u}}-\frac{d}{dS}\hat{g}(\sigma ,F,S)|}^{2}dS}}{{\int }_{0}^{{S}_{u}}|\hat{g}(\sigma ,F,S)|dS}\mathrm{.}$$

Function *D*(*S*_*u*_, σ, *F*) can be interpreted as the weighted “standard deviation” of the *S*-derivative of the diffractogram at given values of σ and *F*. Therefore, decreasing values of function *D*(*S*_*u*_, σ, *F*) in dependence of σ and *F* signal increasing *S*-scaling linearity of $\hat{g}(\sigma ,F,S)$ for 0 ≤ *S* ≤ *S*_*u*_.

**Figure 2:**
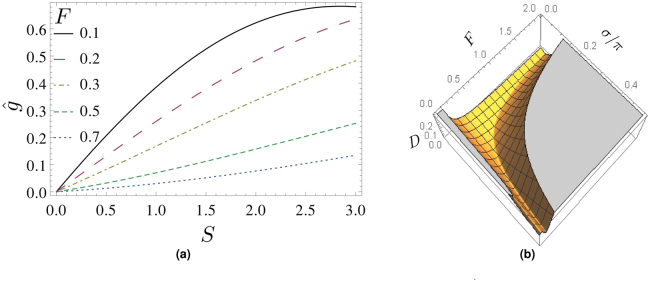
*S*-scaling and valley of linearity. (**a**) Variety of *S*-scaling curves at *σ*/*π* = 0.255 and for *F* = 0.1,0.2,0.3,0.7. Note the transition between convex and concave *S*-scaling at $F\sim 0.3$. (**b**) *D*(*S*_*u*_, *σ*, *F*) of Eq. (11) as a function of *F* and *σ*/*π* at *S*_*u*_ = 1.5. Note the uniqueness of the “floor of the valley” for *σ*/*π* ≤ 0.5.

**Figure 3:**
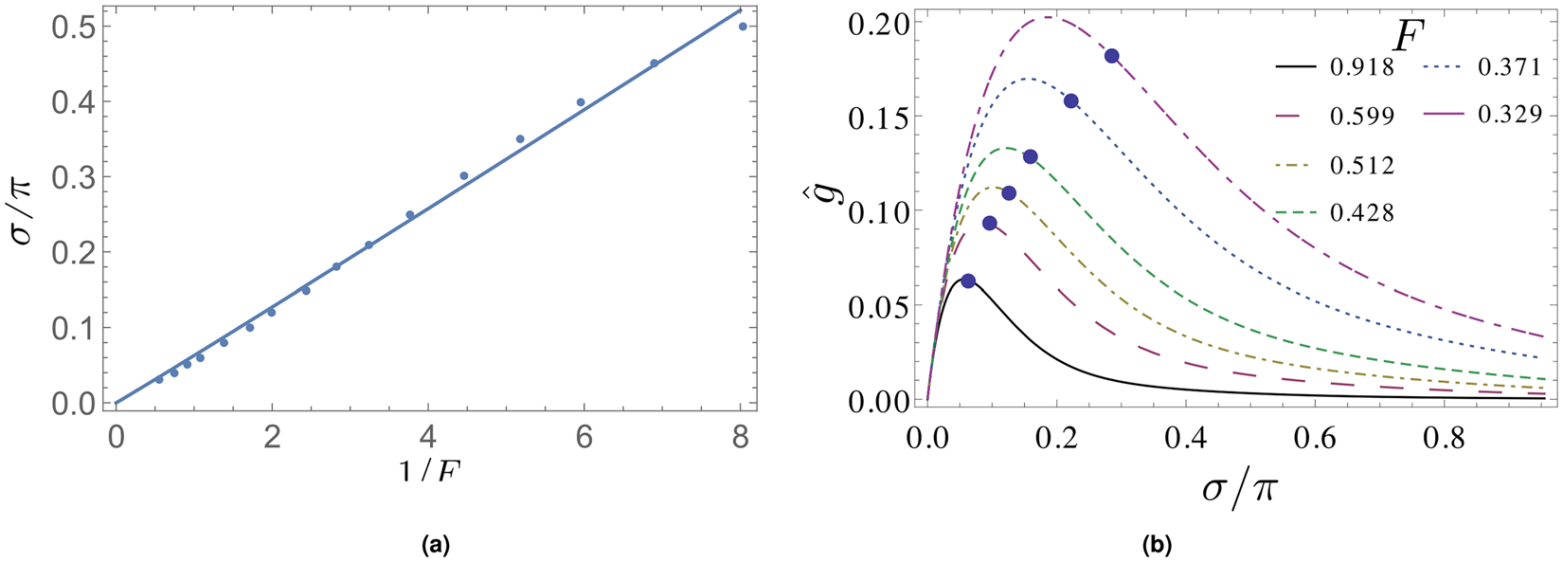
Function *σ*(*F* ^−1^) associated with the floor of the valley in Fig. 2b) and location of *σ*(*F* ^−1^) in the normalized diffractogram $\hat{g}$. (**a**) Plots of *σ*(*F* ^−1^) (dots) and the best-fit to the model (12) (solid line) for *S*_*u*_ = 1.4, compare with Table 1. (**b**) Location of the points *σ*(*F*) in their respective normalized diffractograms $\hat{g}(\sigma ,F,S=1)$. Note that, in contrast to the normalized diffractograms in Fig. 1a, non-oscillatory behavior occurs for these comparably large values of *F*.

The relative error of *D*(*S*_*u*_, σ, *F*) in Fig. 2b never exceeds 10^−6^. Figure 2a shows a variety of *S*-scaling curves, $\hat{g}(\sigma ,F,S)$, for *F* = 0.1, 0.2, 0.3, 0.7 and at σ/π = 0.255. Note the transition from convex to concave *S*-scaling at *F* = 0.3 where linearity in *S* persists longest. In Fig. 2b function *D*(*S*_*u*_, σ, *F*) is depicted in dependence of σ and *F*. Note the occurrence of a valley of *S*-scaling linearity in *F* and σ. The floor of this valley defines a function σ(*F* ^−1^) which is shown in Fig. 3a together with the best-fit to the following power-law (PL) model

12 $$\,{\rm{PL}}({F}^{-1},A,B)=A{({F}^{-1})}^{B}\mathrm{.}$$

Interestingly, parameter *B* shows little deviation from unity for a rather large range of *S*_*u*_ values, see Table 1. Exploiting that $\sigma =\pi w{\xi }^{2}{F}^{-1}$ and setting *B* = 1, we infer that the physical frequency modulus

13 $${\xi }_{{\rm{ml}}}\equiv |\xi {|}_{{\rm{ml}}},$$

where maximal *S*-scaling linearity occurs, is nearly *independent* of *F*. Note that, due to isotropy, *ϕ*(**x**) = *ϕ*(*r*), only the modulus of *ξ* is relevant when searching the region of maximal *S*-scaling linearity. This is expressed by Eq. (13). For example, the value *A* = (6.44 ± 0.07)·10^−2^ at *S*_*u*_ = 1.4 implies

14 $${\xi }_{{\rm{ml}}}=\sqrt{\frac{A}{\pi w}}\sim (0.143\pm 0.001){w}^{-\mathrm{1/2}}\mathrm{.}$$

**Table 1:** Best-fit parameters *A* and *B* for model (12) representing *σ*(*F* ^−1^) in dependence of 1 ≤ *S*_*u*_ ≤ 1.7 and for 0.1 ≤ *F* ≤ 1.5.

*S*_*u*_	*A*	*B*
1.7	(6.20 ± 0.06) ⋅ 10^−2^	1.091 ± 0.006
1.6	(6.30 ± 0.07) ⋅ 10^−2^	1.064 ± 0.007
1.5	(6.37 ± 0.07) ⋅ 10^−2^	1.037 ± 0.008
1.4	(6.44 ± 0.08) ⋅ 10^−2^	1.011 ± 0.007
1.35	(6.48 ± 0.08) ⋅ 10^−2^	0.997 ± 0.007
1.3	(6.53 ± 0.08) ⋅ 10^−2^	0.983 ± 0.007
1.25	(6.55 ± 0.09) ⋅ 10^−2^	0.970 ± 0.008
1	(6.81 ± 0.10) ⋅ 10^−2^	0.900 ± 0.008

Eq. (14) states that, independently of propagation “distance” λ*z*, there exists a single physical frequency modulus *ξ*_ml_ at which the diffractogram *S*-scales in a maximally linear way. The quantitiy *ξ*_ml_ exhibits both the SOBS scale *w* as well as a (presumably shape specific) prefactor. For phase maps controlled by the scaling variable *S* this can be exploited to characterize the according SOBS without the need for phase retrieval: in analogy to the case of Riemannian manifolds of positive sectional curvature the diffractogram possesses a “soul” at *ξ*_ml_^17^.

**Figure 4:**
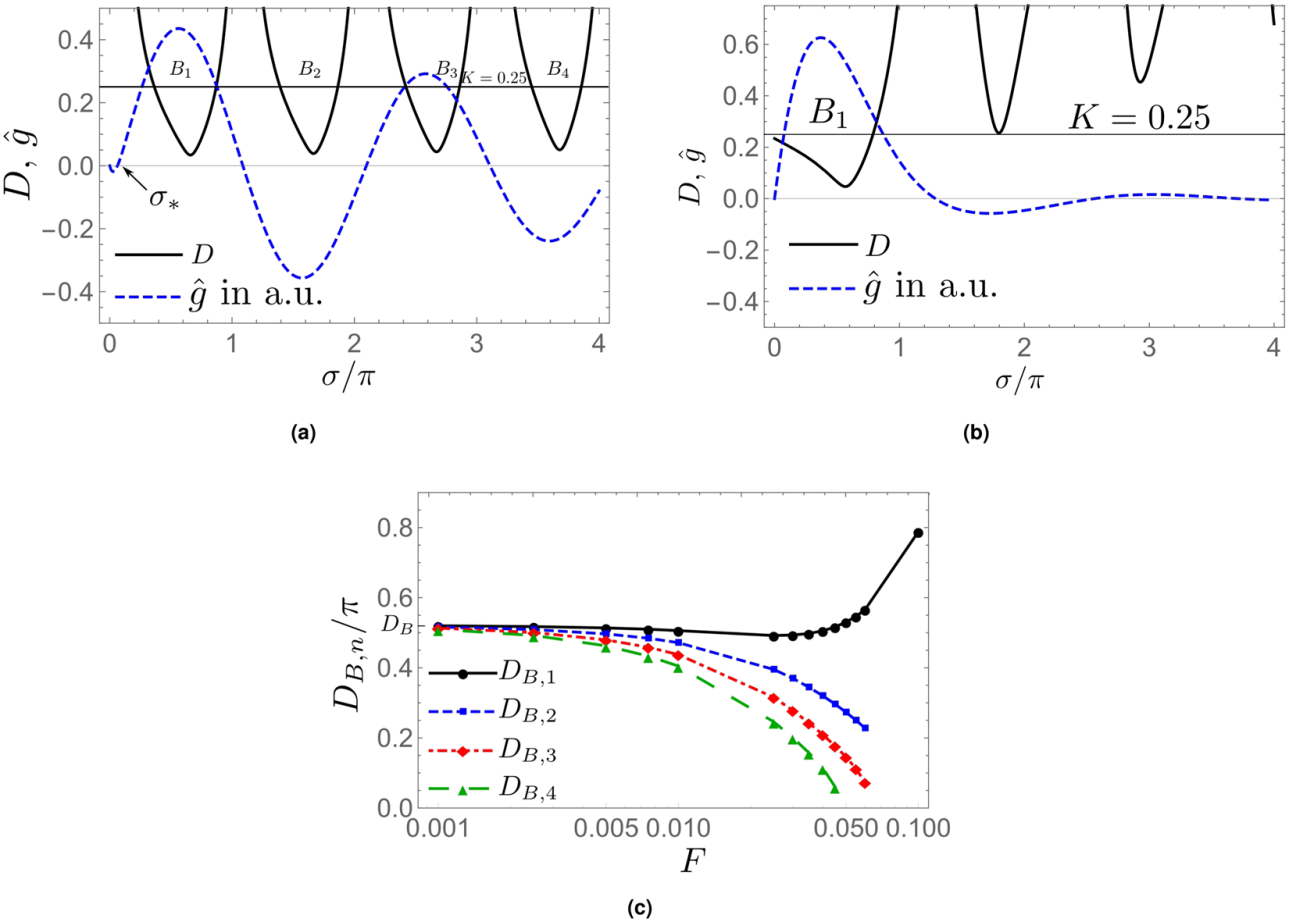
*F* dependence of approximate *S*-scaling linearity (*D* ≤ *K*). (**a**) Damped oscillatory diffractogram (*F* = 0.01, arbitrary units) together with function *D*(*S*_*u*_ = 1.5, *σ*, *F* = 0.01) and *K* = 0.25. Notice the *σ*-bands *B*_1_, …, *B*_4_ where *D* ≤ *K* and the non-linear behavior near *σ* = 0 due to the existence of σ_*_. (**b**) Well-damped diffractogram (*F* = 0.1, arbitrary units) together with function *D*(*S*_*u*_ = 1.5, *σ*, *F* = 0.1) and *K* = 0.25. Notice that only band *B*_1_ exists and now includes *σ* = 0. (**c**) band widths *D*_*B*,*n*_ (*n* = 1, …, 4), see Eq. (17). In contradistinction to *D*_*B*,2_, …, *D*_*B*,4_ notice the increase of *D*_*B*,1_ near *F* = 0.1.

Figure 3b shows, for various values of *F*, that the spectral locations σ(*F*, *S*_max_ = 1.5) in $\hat{g}(\sigma ,F,S=1)$ do not appear to be particularily singled out in the diffractogram. One notes, however, that the oscillatory behavior, encountered in Fig. 1a or Fig. 4a for small values of *F*, no longer is realized in Fig. 3b which is based on comparably large values of *F*. Is the transition from the former to the latter situation a critical phenomenon? This question is addressed in Sec. 2.3.

### Scaling properties of entire diffractogram in dependence of ***F***

After having identified an isolated point of maximum *S*-scaling linearity at low frequencies in Sec. (2.1) we now turn to an investigation of the entire spectrum. In anticipating diffractograms induced by more complex phase maps, see Sec. (2.4), we now understand *S*-scaling linearity collectively in terms of σ-bands *B*_*n*_ (n = 1, 2, 3, …). These bands are defined as

15 $${B}_{n}(F,{S}_{u})\equiv [{\sigma }_{n-1},{\sigma }_{n}]$$

such that

16 $$D({S}_{u},\sigma ,F)\le K,({\sigma }_{n-1}\le \sigma \le {\sigma }_{n}),$$

were *K* > 0. For our subsequent analysis we set *K* = 0.25 and *S*_*u*_ = 1.5. In Fig. 4a a damped oscillatory diffractogram (*F* = 0.01) with *S* = 1 together with the function *D*(*S*_*u*_ = 1.5, σ, *F* = 0.01) are shown for 0 ≤ σ ≤ 4. Also, we depict the line *K* = 0.25. In this situation, we can identify four bands where *D*(*S*_*u*_ = 1.5, σ, *F* = 0.01) ≤ *K*, *B*_1_, …, *B*_4_. Notice the absence of approximate *S*-scaling linearity near σ = 0 due to the existence of the additional zero σ_*_, discussed in Sec. 1. Figure 4b shows the well-damped case at *F* = 0.1. Notice that only the band *B*_1_ survives under the change *F* = 0.01 to *F* = 0.1 and now includes σ = 0. This process is characterized by Fig. 4c where the bandwidth *D*_*B,n*_, defined as

17 $${D}_{B,n}\equiv {\sigma }_{n}-{\sigma }_{n-1},$$

is depicted as a function of *F*. For *F* → 0 the *D*_*B,n*_ are universal, *D*_*B,n*_ ≡ *D*_*B*_. On the other hand, monotonic shrinkage and eventual disappearance of *B*_2_, *B*_3_, … as well as the non-monotonic behavior of *D*_*B*,1_ occur for increasing *F*. Indeed, *D*_*B*,1_ increases for *F* > 0.025 which implies that the diffractogram *S*-scales most linearly at low frequencies. This linearity, however, is not to be confused with CTF-like linear phase-to-intensity contrast transfer: it emerges due to strong non-local contributions in the onion-shell expansion, see Sec. 3.1.

### Transition from damped oscillatory to non-oscillatory diffractogram

**Figure 5:**
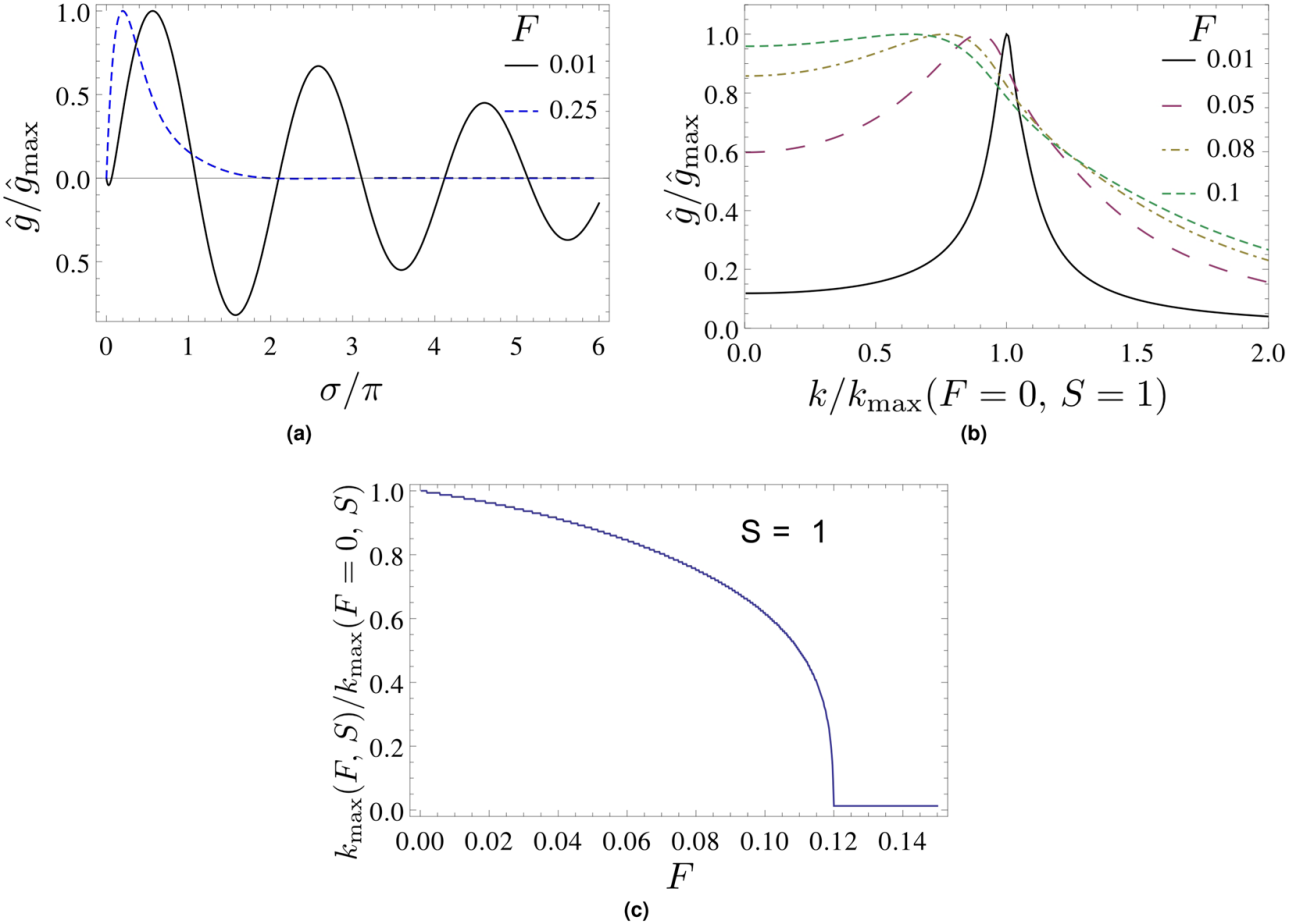
Global transition in normalized diffractogram as a function of *F* at *S* = 1. (**a**) Damped oscillatory (*F* = 0.01) and overdamped non-oscillatory (*F* = 0.25) behavior. (**b**) Positive-*k* branch of $\tilde{g}$, for definition see Eq. (18). (**c**) Normalized *k*_max_(*F*, *S* = 1), for definition see text. Note the critical drop at *F* (*S* = 1) = 0.12 (associated with a critical exponent *ν*(*S* = 1) = 0.37).

We would now like to discuss an *F*-dependent transition affecting the *entire* diffractogram. Figure 5a indicates the nature of this transition: a damped oscillatory diffractogram converts into an overdamped non-oscillatory one under an increase of *F* at *S* = 1. Notice that the (pseudo) frequency *k* or the period of the damped oscillation (inverse of twice the distance between adjacent zero crossings in $\hat{g}(\sigma ,F,S)$) is constant throughout the entire diffractogram. To judge whether this transition is a critical phenomenon, we define *k*_max_(*F*, *S*) to be the peak position in the positive-*k* branch of the 1D Fourier transform $\tilde{g}(k,F,S)$ of $\hat{g}(\sigma ,F,S)$ w.r.t. σ:

18 $$\tilde{g}(k,F,S)\equiv {\int }_{-\infty }^{\infty }{\rm{d}}\sigma \,\exp (2\pi {\rm{i}}k\sigma )\hat{g}(\sigma ,F,S),$$

see Fig. 5b. Figure 5c indicates that *k*_max_(*F*, *S* = 1) behaves like an order parameter of a second-order phase transition with the critical drop at *F* (*S* = 1) = 0.12, exhibiting an exponent of *v* (*S* = 1) = 0.37. We mention in passing that *F* (*S* = 0.5) = 0.13, *v* (*S* = 0.5) = 0.51 and *F* (*S* = 1.5) = 0.11, *v* (*S* = 1.5) = 0.27. Therefore, the transition occurs at decreasing *F* for increasing *S*, and an according critical phenomenon occurs at fixed *F* in dependence on *S*, for more details see^18^.

### Outlook on selected multi-scale objects of a broad spectrum (MOBS)

The case of a Gaussian SOBS phase map obviously is a strong simplification, and the question naturally arises whether certain aspects of the observations made in Secs. 2.2 and 2.3 are generic. Here, we perform an analysis of the scaling behavior of a *simulated* diffractogram, based on the Lena phase map, where bands of approximate *S*-scaling linearity are observed whose width evolution does not behave in the same ordered way seen in Fig. 4c. Moreover, the non-linearity at small σ and small *F* in the case of the Gaussian SOBS phase map no longer occurs in the simulated diffractograms at large *z*. This is expressed by a considerably larger band width *D*_*B*,1_ compared to *D*_*B*,*n*>1_ for all *z*. Moreover, we give experimental evidence for the transition seen in Sec. 2.3 from a damped oscillatory to an overdamped non-oscillatory diffractogram, as induced by a test sample, depending on propagation distance *z*.

**Figure 6:**
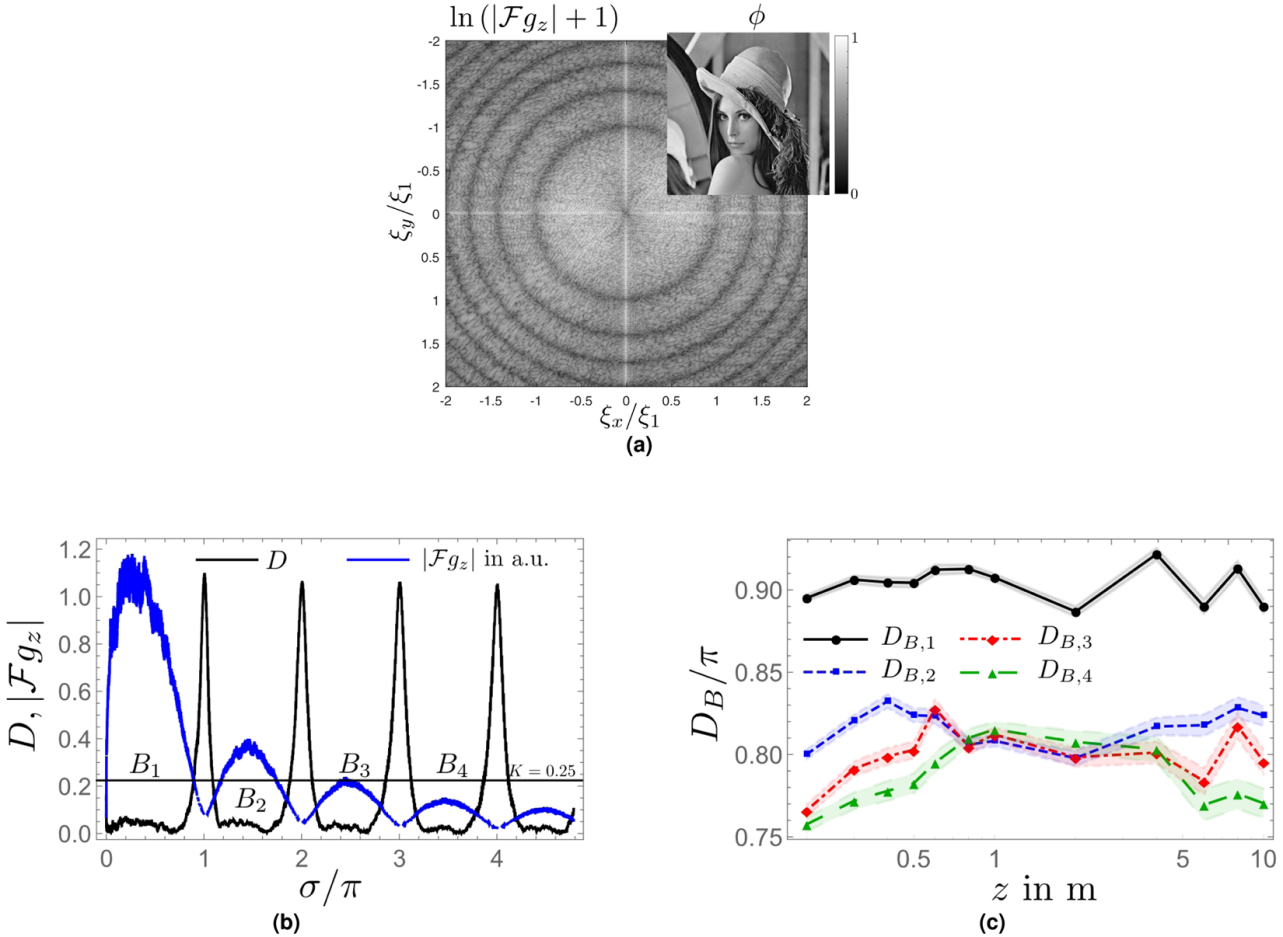
Simulated diffractogram as induced by Lena phase map for *E* = 1 keV and a pixel size Δ*x* = 1.6*μ*m (1024 × 1024 pixel). (**a**) Lena phase-map and 2D “diffractogram” *ln*(1 + |*Fg*_*z*_|) at *z* = 1 m. The first minima is at |*ξ*| = *ξ*_1_. (**b**) Angular-averaged diffractogram (arbitrary units) at *z* = 0.2 m and (discretized version of) function *D* in Eq. (11). (**c**) Widths of linearity bands, *D*_*B*,*n*_ (*n* = 1, …, 4), for definition see Eq. (17, in dependence of *z*. Shaded error bands indicate uncertainties due to discretization, see^18^.

Figure 6a depicts the 2D diffractogram as induced by the (positive definite) Lena phase map, normalized to a maximum of unity, Fig. 6b shows the angular average of the diffractogramm together with a discretized version of function *D* (see Eq. (11)), measuring σ dependent *S*-scaling linearity, and Fig. 6c indicates an approximate *z* independence of the widths of linearity bands in contrast to the behavior shown in Fig. 4c.

**Figure 7:**
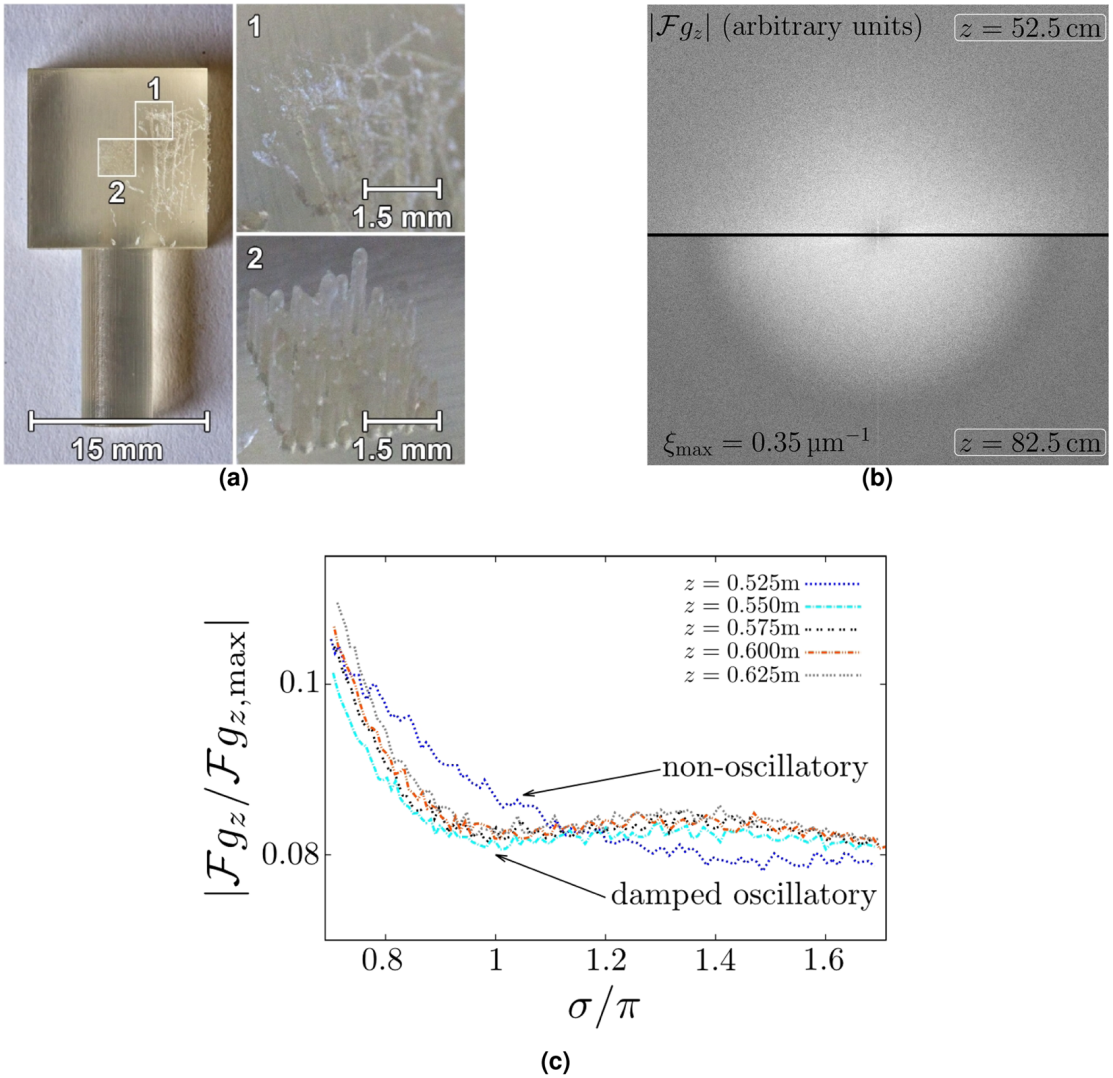
ESRF experiment at beamline ID19^15^ with *E* = 19.4 keV, Δ*E*/*E* = 0.03, and effective pixel size Δ*x* = 1.6*μ* m. The physical pixel size was 6.4 *μ* m, necessitating a 4x magnifying lense system for visible light. Assuming a maximum structure thickness of Δ*z* = 0.02 mm of the scratched region 1, see (**a**) below, and a real decrement *δ* = 5.66 × 10^−7^ (refractive index of propylene at *E* = 19.4 keV), we estimate the maximal phase shifts $\frac{2\pi \delta }{\lambda }{\rm{\Delta }}z$ to be of order unity as in Fig. 5. On the other hand, assuming the smallest spatial scale in region 1 to be given by the resolution limit 2Δ*x*, the associated Fresnel number is 0.27 which is comparable to the transition value of *F* in Fig. 5. The camera employed was a pco.edge behind a 10 *μ* m thick GGG:Eu scintillator. The exposure time of *τ* = 50 ms corresponded to a mean count number of $\sim 9500$. (**a**) 3D printed pure-phase sample. Only data associated with region 1 is analyzed here, for details see^15^. (**b**) Diffractograms at *z* = 0.525 m (upper) and *z* = 0.825 m (lower), (**c**) Normalized, angular-averaged diffractogram as induced by region 1 for various values of *z*.

Figure 7a is a photograph of the (nearly) pure-phase sample: a 3D printed rectangular solid with a scratched surface (region 1). In Fig. 7b diffractograms induced by the X-ray projection of this sample and subsequent parallel-beam free-space propagation are depicted for two propagation distances. Both the maximum phase shift and the Fresnel number associated with the resolution limit are comparable to the values used in Fig. 5 for the Gaussian SOBS case. Notice a qualitative structural difference between the two diffractograms. Figure 7c shows a family of normalized, angular-averaged diffractograms. Here, the transition from damped oscillatory diffractograms ($z\ge 0.550\,$ m) and to the overdamped non-oscillatory diffractogram at *z* = 0.525 m is clearly seen. This sharp transition is excluded to be due to imperfect temporal coherence^15^ and thus resembles the transition seen in Fig. 5 for the Gaussian SOBS case.

## The extreme far-field

### SOBS: Examples for σ–universality of diffractograms

Here, we isolate universal aspects of the extreme far-field behavior within SOBS induced diffractograms. Recall, that in this regime the diffractogram is well-dominated by the zeroth onion-shell contribution (*l* = 0). To make the point exemplarily, let us consider normalized diffractograms as induced by three different SOBS phase maps: (i) circular-disk, (ii) exponential, and (iii) Gaussian phase. For a given phase map *ϕ* the zeroth onion-shell contribution ${\mathcal F} {g}_{z\mathrm{,0}}$ reads^15^

19 $$ {\mathcal F} {g}_{z\mathrm{,0}}=2\,\sin (\sigma )\sum _{k=0}^{\infty }\frac{{(-1)}^{k} {\mathcal F} {{\varphi }}^{2k+1}}{\mathrm{(2}k+\mathrm{1)!}}+2\,\cos (\sigma )\sum _{k=1}^{\infty }\frac{{(-1)}^{k} {\mathcal F} {{\varphi }}^{2k}}{(2k)!}.$$

Concerning case (i), the phase map *ϕ* is defined as

20 $${\varphi }(r)=S\,\theta (\sqrt{w}-r),$$

where *θ*(*x*) denotes the Heaviside step function and *r* ≡ |**x**_⊥_|. In the limit *F* ≡ *w*/(*λz*) → 0 and for σ > 0 Eq. (19) represents the diffractogram is exactly. In substituting Eq. (20) into Eq. (19), one arrives at

$$\mathop{\mathrm{lim}}\limits_{F\to 0} {\mathcal F} {g}_{z}=\mathop{\mathrm{lim}}\limits_{F\to 0} {\mathcal F} {g}_{z\mathrm{,0}}\equiv \mathop{\mathrm{lim}}\limits_{F\to 0}[ {\mathcal F} {g}_{z,\sin \mathrm{,0}}+ {\mathcal F} {g}_{z,\cos \mathrm{,0}}]\,(\sigma  > 0),$$

where

21 $$\begin{array}{rcl} {\mathcal F} {g}_{z,\sin \mathrm{,0}} & = & 2\,\sin (\sigma )\sum _{k=0}^{\infty }\frac{{(-1)}^{k}}{(2k+1)!} {\mathcal F} {S}^{2k+1}{\theta }^{2k+1}(\sqrt{w}-r)\\  & = & 4\pi w\,\sin (\sigma )\sin (S)\sqrt{\pi }\frac{{J}_{1}(2\sqrt{\pi \sigma F})}{\sqrt{\sigma F}},\end{array}$$

and where

22 $$\begin{array}{rcl} {\mathcal F} {g}_{z,\cos \mathrm{,0}} & = & 2\,\cos (\sigma )\sum _{k=1}^{\infty }\frac{{(-1)}^{k}}{(2k)!} {\mathcal F} {S}^{2k}{\theta }^{2k}(\sqrt{w}-r)\\  & = & 4\pi w\,\cos (\sigma )(\cos (S)-1)\,\sqrt{\pi }\,\frac{{J}_{1}(2\sqrt{\pi \sigma F})}{\sqrt{\sigma F}}.\end{array}$$

In deriving Eqs (21) and (22) we have used that *θ* ^*n*^(*x*) = *θ*(*x*) (*n* = 1, 2, …) with the exception of point *x* = 0 which has measure zero in the Fourier-transform integral. In Eqs (21) and (22) the Bessel function *J*_1_ of the first kind occurs. It is defined via the power series

23 $${J}_{1}(x)\equiv \sum _{m=0}^{\infty }\frac{{(-1)}^{m}}{m!{\rm{\Gamma }}(m+2)}{(\frac{x}{2})}^{2m+1},$$

where Γ denotes the gamma function. Therefore, we have

24 $$\begin{array}{rcl}\mathop{\mathrm{lim}}\limits_{F\to 0}\frac{ {\mathcal F} {g}_{z\mathrm{,0}}}{4\pi w} & = & \mathop{\mathrm{lim}}\limits_{F\to 0}(\sin (\sigma )\sin (S)+\,\cos (\sigma )(\cos (S)-1))\cdot \,\sqrt{\pi }\frac{{J}_{1}\mathrm{(2}\sqrt{\pi \sigma F})}{\sqrt{\sigma F}}\\  & = & \pi (\sin (\sigma )\sin (S)+\,\cos (\sigma )(\cos (S)-1)).\end{array}$$

Case (ii) is based on the following phase map

25 $${\varphi }(r)=S\,\exp (-\frac{r}{\sqrt{w}}).$$

According to Eq. (19) the contributions to the zeroth onion shell read

26 $$\begin{array}{ll} {\mathcal F} {g}_{z,\sin \mathrm{,0}} & =2\,\sin (\sigma )\sum _{k=0}^{\infty }\frac{{(-1)}^{k}}{(2k+1)!} {\mathcal F} {S}^{2k+1}\exp (-\frac{r}{\sqrt{w}}(2k+1))\\  & =4\pi w\,\sin (\sigma )\sum _{k=0}^{\infty }\frac{{(-1)}^{k}{S}^{2k+1}(2k+1)}{(2k+1)!{({(2k+1)}^{2}+4\pi \sigma F)}^{\mathrm{3/2}}}\,\end{array}$$

and

27 $$\begin{array}{rcl} {\mathcal F} {g}_{z,\cos \mathrm{,0}} & = & 2\,\cos (\sigma )\sum _{k=1}^{\infty }\frac{{(-1)}^{k}}{\mathrm{(2}k)!} {\mathcal F} {S}^{2k}\exp (-\frac{r}{\sqrt{w}}2k)\\  & = & 4\pi w\,\cos (\sigma )\sum _{k=1}^{\infty }\frac{{(-1)}^{k}{S}^{2k}2k}{(2k)!{(4{k}^{2}+4\pi \sigma F)}^{\mathrm{3/2}}}.\end{array}$$

Adding Eqs (26) and (27), one obtains

28 $$\begin{array}{ll}\mathop{\mathrm{lim}}\limits_{F\to 0}\frac{ {\mathcal F} {g}_{z\mathrm{,0}}}{4\pi w} & =\mathop{\mathrm{lim}}\limits_{F\to 0}[\sin (\sigma )\sum _{k=0}^{\infty }\frac{{(-1)}^{k}{S}^{2k+1}(2k+1)}{(2k+1)!{({\mathrm{(2}k+1)}^{2}+4\pi \sigma F)}^{\mathrm{3/2}}}+\,\cos (\sigma )\sum _{k=1}^{\infty }\frac{{(-1)}^{k}{S}^{2k}2k}{(2k)!{(4{k}^{2}+4\pi \sigma F)}^{\mathrm{3/2}}}]\\  & =\,\sin (\sigma )\sum _{k=0}^{\infty }\frac{{(-1)}^{k}{S}^{2k+1}}{(2k+1)!{(2k+1)}^{2}}+\,\cos (\sigma )\sum _{k=1}^{\infty }\frac{{(-1)}^{k}{S}^{2k}}{(2k)!{(2k)}^{2}}.\end{array}$$

Finally, case (iii) can be inferred from Eq. (D12) in^15^. One obtains

29 $$\begin{array}{rcl}\mathop{\mathrm{lim}}\limits_{F\to 0}\frac{ {\mathcal F} {g}_{z\mathrm{,0}}}{4\pi w} & = & \mathop{\mathrm{lim}}\limits_{F\to 0}[\sin (\sigma )\sum _{k=0}^{\infty }\frac{{S}^{2k+1}}{2k+1}\frac{{(-1)}^{k}\exp (-\sigma \frac{2\pi F}{2k+1})}{(2k+1)!}\\  &  & +\,\cos (\sigma )\sum _{k=1}^{\infty }\frac{{S}^{2k}}{2k}\frac{{(-1)}^{k}\exp (-\sigma \frac{2\pi F}{2k})}{(2k)!}]\\  & = & \sin (\sigma )\sum _{k=0}^{\infty }\frac{{S}^{2k+1}}{2k+1}\frac{{(-1)}^{k}}{(2k+1)!}+\,\cos (\sigma )\sum _{k=1}^{\infty }\frac{{S}^{2k}}{2k}\frac{{(-1)}^{k}}{(2k)!}\,\mathrm{.}\end{array}$$

Let us now introduce the normalization *N*_*w*_ such that

30 $$\mathop{\mathrm{lim}}\limits_{w\to 0}{{N}_{w}^{-1}{\varphi }(r)|}_{S=1}={\delta }^{\mathrm{(2)}}({\bf{x}}),$$

where *δ* ^(2)^(**x**) denotes the 2D Dirac delta function. For (i), (ii), and (iii) one obtains *N*_*w*_ = *πw*, *N*_*w*_ = 2*πw*, and *N*_*w*_ = 2*πw*, respectively. Defining the general, normalized SOBS-diffractogram in the extreme far-field limit as

31 $$\begin{array}{cc}\mathop{{\rm{l}}{\rm{i}}{\rm{m}}}\limits_{F\to 0}\frac{{\mathscr{F}}{g}_{z}}{{N}_{w}} & =\,2\,\sin (\sigma )\sum _{k=0}^{{\rm{\infty }}}\frac{{(-1)}^{k}}{(2k+1)!}{S}^{2k+1}C(2k+1)+2\,\cos (\sigma )\sum _{k=1}^{{\rm{\infty }}}\frac{{(-1)}^{k}}{(2k)!}{S}^{2k}C(2k)\\  & \equiv \,{{\mathscr{S}}}_{s}(S)\sin (\sigma )+{{\mathscr{S}}}_{c}(S)\cos (\sigma )(\sigma  > 0),\end{array}$$

we read off from the last lines in Eqs (24), (28), and (29) that

32 $$C(j)=\{\begin{array}{cc}2\pi  & {\rm{c}}{\rm{i}}{\rm{r}}{\rm{c}}{\rm{u}}{\rm{l}}{\rm{a}}{\rm{r}}\,{\rm{d}}{\rm{i}}{\rm{s}}{\rm{k}},\,{\rm{c}}{\rm{a}}{\rm{s}}{\rm{e}}\,({\rm{i}}),\\ {j}^{-2} & \,{\rm{e}}{\rm{x}}{\rm{p}}{\rm{o}}{\rm{n}}{\rm{e}}{\rm{n}}{\rm{t}}{\rm{i}}{\rm{a}}{\rm{l}},\,{\rm{c}}{\rm{a}}{\rm{s}}{\rm{e}}\,({\rm{i}}{\rm{i}})\,,\\ {j}^{-1} & {\rm{G}}{\rm{a}}{\rm{u}}{\rm{s}}{\rm{s}}{\rm{i}}{\rm{a}}{\rm{n}},\,{\rm{c}}{\rm{a}}{\rm{s}}{\rm{e}}\,({\rm{i}}{\rm{i}}{\rm{i}}).\end{array}$$

According to Eq. (32), we may state that the phase-shape information, which is different for cases (i), (ii), and (iii), transmutes into information residing in the scaling functions ${{\mathscr{S}}}_{c}(S)$, ${{\mathscr{S}}}_{s}(S)$ of Eq. (31): Their coefficients C(*j*) in Eq. (32) are specific for each of the three cases. Except for this specifity in *S*-scaling the diffractogram is σ-universal in the extreme far-field: its σ dependence is represented by sine and cosine functions. This σ-universality was noticed already in^16^.

### MOBS: Transmutation of phase-shape to *S*–scaling information

The observation made in the last section that SOBS-phase shape information transmutes into *S*-scaling information in the extreme far-field limit can be generalized to those MOBS phase maps ${{\varphi }}_{\{{w}_{i}\}}$, carrying spatial scales {*w*_*i*_}, for which the following condition holds: There exists a normalization ${N}_{\{{w}_{i}\}}$ such that

33 $$\mathop{\mathrm{lim}}\limits_{\kappa \to 0}{{N}_{\{\kappa {w}_{i}\}}^{-1}{{\varphi }}_{\{\kappa {w}_{i}\}}({x})|}_{S=1}={\delta }^{\mathrm{(2)}}({{x}}_{\perp }).$$

Obviously, (33) generalizes (30). To show far-field σ-universality under condition (33), we write

34 $${{\varphi }}_{\{{w}_{i}\}}({{\bf{x}}}_{\perp })=S{N}_{\{{w}_{i}\}}{\delta }_{\{{w}_{i}\}}({\bf{x}}),$$

with

35 $$\mathop{\mathrm{lim}}\limits_{\kappa \to 0}{\delta }_{\{\kappa {w}_{i}\}}({\bf{x}})={\delta }^{\mathrm{(2)}}({\bf{x}}).$$

Eq. (19) demands the evaluation of ${\mathcal F} {{\varphi }}_{\{\kappa {w}_{i}\}}^{j}$ for *j* = 1, 2, …. Recall that, except for σ = 0, the zeroth onion-shell approximation ${\mathcal F} {g}_{z}\sim  {\mathcal F} {g}_{z\mathrm{,0}}$ is exact for $\kappa \to 0$. Appealing to the Fourier convolution theorem, we have

36 $$\mathop{\mathrm{lim}}\limits_{\kappa \to 0} {\mathcal F} {{\varphi }}_{\{\kappa {w}_{i}\}}^{j}({{\bf{x}}}_{\perp })={S}^{j}\times \mathop{\mathrm{lim}}\limits_{\kappa \to 0}{N}_{\{\kappa {w}_{i}\}}{N}_{\{\kappa {w}_{i}\}}^{j-1}\mathop{\underbrace{ {\mathcal F} {\delta }_{\{\kappa {w}_{i}\}}({{\bf{x}}}_{\perp })\ast \mathrm{...}\ast  {\mathcal F} {\delta }_{\{\kappa {w}_{i}\}}({{\bf{x}}}_{\perp })}}\limits_{j-1\,{\rm{convolutions}}}.$$

Let us define the dimensionless quantity $W(j-1,\{\kappa {w}_{i}\})$ as

37 $$W(j-\mathrm{1,\{}\kappa {w}_{i}\})\equiv {N}_{\{\kappa {w}_{i}\}}^{j-1}\mathop{\underbrace{ {\mathcal F} {\delta }_{\{\kappa {w}_{i}\}}({{\bf{x}}}_{\perp })\ast \mathrm{...}\ast  {\mathcal F} {\delta }_{\{\kappa {w}_{i}\}}({{\bf{x}}}_{\perp })}}\limits_{j-1\,{\rm{convolutions}}}.$$

By comparison with the analogue of Eq. (31) we identify

38 $$C(j)=\mathop{\mathrm{lim}}\limits_{\kappa \to 0}W(j-\mathrm{1,\{}\kappa {w}_{i}\mathrm{\}).}$$

In Eq. (38) the dependence of C(*j*) on the dimensionless ratios ${w}_{i}/{w}_{i^{\prime} }(i > i^{\prime} )$ is notationally suppressed. Thus, the extreme far-field limit of the normalized diffractogram reads

39 $$\begin{array}{cc}\mathop{{\rm{l}}{\rm{i}}{\rm{m}}}\limits_{\kappa \to 0}\frac{{\mathscr{F}}{g}_{z}}{{N}_{\{\kappa {w}_{i}\}}} & =2(\sin (\sigma )\sum _{k=0}^{{\rm{\infty }}}\frac{{(-1)}^{k}{S}^{2k+1}C(2k+1)}{(2k+1)!}+\cos (\sigma )\sum _{k=1}^{{\rm{\infty }}}\frac{{(-1)}^{k}{S}^{2k}C(2k)}{(2k)!})\\  & \equiv \,{{\mathscr{S}}}_{s}(S)\,\sin (\sigma )+{{\mathscr{S}}}_{c}(S)\,\cos (\sigma )\,(\sigma  > 0).\end{array}$$

In the extreme far-field limit Eq. (39) represents the generalization of certain SOBS-phase induced diffractograms to certain MOBS-phase induced diffractograms. Again, the limit ${F}_{\kappa {w}_{i}}\to 0$ transmutes phase-shape information into scaling information captured by functions ${{\mathscr{S}}}_{c}(S),{{\mathscr{S}}}_{s}(S)$. That this transmutation is *not* injective is demonstrated by two example diffractograms induced by

40 $$\begin{array}{ll}\,({\rm{i}}):\,{\varphi } & =S\,\exp (-\frac{{x}^{2}+{\rm{\Omega }}{y}^{2}}{2w})\,,\\ \,({\rm{ii}}):\,{\varphi } & =\frac{S}{2}[\exp (-\frac{{{\bf{x}}}^{2}}{2w})+\exp (-\frac{{\rm{\Omega }}{{\bf{x}}}^{2}}{2w})]\,\mathrm{.}\end{array}$$

Accordingly, we derive from Eqs. (37) and (38):

41 $$\begin{array}{cc}\,({\rm{i}}):\,{N}_{w,w/{\rm{\Omega }}}=2\pi \frac{w}{\sqrt{{\rm{\Omega }}}}\,,\,\,\,\,\,\,\,\,\,\,\, & C(j)={j}^{-1}\\ \,({\rm{i}}{\rm{i}}):\,{N}_{w,w/{\rm{\Omega }}}=\pi w(1+{{\rm{\Omega }}}^{-1})\,,\,\, & C(j)=\frac{{2}^{-j+1}}{1+{{\rm{\Omega }}}^{-1}}\sum _{l=0}^{j}(\begin{array}{c}j\\ l\end{array})\frac{1}{j+l({\rm{\Omega }}-1)}\,.\end{array}$$

Note that in Eq. (41) the case (i) yields the same coefficient *C*(*j*) = *j* ^−1^ as in case (iii) of Eq. (32). It is conceivable that Eq. (39) has practical implications if phase maps can be scaled physically, e.g. by increase of the refractive index along the direction of projection through a phase sample by virtue of external electromagnetic fields.

## Discussion

In this work we have investigated the *S*-scaling properties of diffractograms induced by free-space propagation of wave fields subject to a Gaussian phase map of width $\sqrt{w}$. In particular, a value of the (dimensionful) frequency modulus, *ξ*_ml_ = (0.143 ± 0.001)*w* ^−1/2^, was found to exist where *S*-scaling is maximally linear. Moreover, for small values of *F* (far-field situation) we have observed the occurrence of several σ-bands of approximate *S*-scaling linearity, none of which includes the point σ = 0. On the contrary, the near-field case (large values of *F*) exhibits a single σ-band only which now contains the point σ = 0. We also have analyzed a simulated diffractogram, based on a Lena phase map, to compare results on *S*-scaling linearity. The Gaussian-phase diffractogram was also shown to critically undergo a change of its behavior in dependence of Fresnel number *F*. Namely, a sharp transition from damped oscillatory to overdamped non-oscillatory was identified for increasing *F*. As a function of propagation distance *z* such a transition is also observed in experimental diffractograms induced by a more complex test sample. Finally, we have noticed a transmutation of phase-shape to *S*-scaling information in the extreme far-field situation for diffractograms induced by three different SOBS phase maps. This result is generalizable to extreme far-field diffractograms as induced by certain multi-scale phase maps.

Practical exploitations of our far-field results for SOBS phases would determine the shape and the scale $\sqrt{w}$ of the SOBS by directly fitting the coefficients *C*(*j*) and *w* to the far-field expression of Eq. (31) at convenient values of σ. This presumes the *S*-scalability of the pure phase object, e.g. in terms of a homogeneous electric field^19^ or an astrophysically induced scaling of the object to be analyzed (e.g. time-dependent gravitational lensing, pulsating sources).

The physics implications of our present work are two-fold. First, for the single-scale case we characterize diffractograms in dependence of *S* and *F* towards the identification of linear regions (points) such that the inducing phase is already revealed in the diffractogram. Second, there should be interesting (critical) diffractive phenomena in dependence of the phase maps’s complexity. For few-scale objects the definition of complexity is straight-forward: a counting of and a classification of the hierarchy of the spatial scales involved. For many-scale objects the notion of an entropy of the phase map is in order. Based on our present results, our expectation is that with increased complexity there are critical transitions such that diffractograms are more and more dominated by linear bands (emergent linearity). The constancy of the band widths suggest a physical regularization of CTF based phase retrieval, so-called QP phase retrieval^13^. To investigate this in detail is important to identify the limits of linear approximations as phase-map complexity *decreases*, thereby allowing experimental or observational conditions to be tuned towards genuinely quantitative phase retrieval.
